# Epidemics Modelings: Some New Challenges

**DOI:** 10.1063/1.3498081

**Published:** 2010-09-30

**Authors:** Stefanella Boatto, Renata Stella Khouri, Lucas Solerman, Claudia Codeço, Catherine Bonnet

**Affiliations:** a Departamento de Matemática Aplicada, Instituto de Matemática, CCMN, Universidade Federal de Rio de Janeiro, Brazil; b FIOCRUZ, Rio de de Janeiro, Brazil; c INRIA‐Saclay, France; University of Peloponnese; University of Buckingham; Technological Educational Institution of Chalkis

## Abstract

Epidemics modeling has been particularly growing in the past years. In epidemics studies, mathematical modeling is used in particular to reach a better understanding of some neglected diseases (dengue, malaria, …) and of new emerging ones (SARS, influenza A,….) of big agglomerates. Such studies offer new challenges both from the modeling point of view (searching for simple models which capture the main characteristics of the disease spreading), data analysis and mathematical complexity. We are facing often with complex networks especially when modeling the city dynamics. Such networks can be static (in first approximation) and homogeneous, static and not homogeneous and/or not static (when taking into account the city structure, micro‐climates, people circulation, etc.). The objective being studying epidemics dynamics and being able to predict its spreading.

